# Bacterial community responses to micropollutants in chemically stressed small rivers in Kenya using environmental DNA

**DOI:** 10.1093/femsle/fnaf113

**Published:** 2025-10-16

**Authors:** Nicolai Verbücheln, Sonja Schaufelberger, Tibaud Cardis, Isaac C Tanui, Faith Kandie, Werner Brack, Thomas Backhaus, Pedro A Inostroza

**Affiliations:** Department of Biological and Environmental Sciences, University of Gothenburg, 41390 Gothenburg, Sweden; Department of Marine Sciences, University of Gothenburg, 41390 Gothenburg, Sweden; Department of Biological and Environmental Sciences, University of Gothenburg, 41390 Gothenburg, Sweden; Department of Marine Sciences, University of Gothenburg, 41390 Gothenburg, Sweden; Department of Exposure Science, Helmholtz Centre for Environmental Research (UFZ), 04318 Leipzig, Germany; Institute of Ecology, Evolution and Diversity—Goethe University, 60438 Frankfurt am Main, Germany; Department of Chemistry and Biochemistry, Moi University, 3900-30100 Eldoret, Kenya; Department of Biological Sciences, Moi University, 3900-30100 Eldoret, Kenya; Stellenbosch Institute for Advanced Study, 7600 Stellenbosch, South Africa; Department of Exposure Science, Helmholtz Centre for Environmental Research (UFZ), 04318 Leipzig, Germany; Institute of Ecology, Evolution and Diversity—Goethe University, 60438 Frankfurt am Main, Germany; Department of Biological and Environmental Sciences, University of Gothenburg, 41390 Gothenburg, Sweden; Institute for Environmental Research, RWTH Aachen University, 52074 Aachen, Germany; Department of Biological and Environmental Sciences, University of Gothenburg, 41390 Gothenburg, Sweden; Institute for Environmental Research, RWTH Aachen University, 52074 Aachen, Germany

**Keywords:** microbial ecotoxicology, micropollutant, eDNA, land use, nutrients, antimicrobial

## Abstract

The responses of bacterial communities to changing environmental conditions are manifold but can include structural as well as functional alterations depending on the environmental stressors and toxic chemicals they are exposed to (e.g. pharmaceuticals, personal care products, pesticides, and industrial chemicals). In this study, environmental DNA was extracted from surface water samples collected from four small rivers in the Lake Victoria South Basin (Western Kenya) to (i) evaluate whether alpha- and beta-diversity change in dependency of land-use types, (ii) identify the environmental variables that explain alterations in community structure, (iii) qualitatively and quantitatively assess the consequences of antimicrobial stress on bacterial communities, and (iv) evaluate bacterial functional changes related to the degradation of organic chemicals. Our findings suggest that bacterial community composition is a more sensitive indicator to reflect the impact of chemical pollution derived from different types of land use compared to alpha-diversity. Nutrients and stress from chemical pollution were the variables explaining the dissimilarities between bacterial communities in small, forested, urbanised, and agricultural rivers. Furthermore, an assessment of potential ecological functions associated with the biodegradation of toxic chemicals unveiled a season-specific decline in bacterial degradation potential in all four rivers.

## Teaser

Bacterial community composition in Lake Victoria South Basin rivers proved a more sensitive indicator of chemical pollution from varying land use than alpha-diversity, with nutrients and chemical stress explaining community differences and biodegradation potential showing seasonal declines.

## Introduction

Rivers and streams are currently among the most altered ecosystems on Earth, which is a consequence of the ever-increasing pressure from anthropogenic activities. Chemical pollutants represent one of the main drivers impacting aquatic biodiversity (Sigmund et al. 2023) due the high toxicity of many environmentally relevant chemicals, their widespread co-occurrence and pseudo-persistence in the aquatic environment. These substances are released into the aquatic environment from various sources including agricultural and urban runoff as well as untreated and treated sewage (Reiber et al. 2021, Soriano et al. 2024, Tanui et al. 2024). Although they are detected at low concentrations (pg/L to ng/L), thus called micropollutants or contaminants of emerging concern (Schwarzenbach et al. 2006, Bertram et al. 2022), these compounds and their metabolites are often bioactive and their continuous release into the aquatic environment makes them “pseudo-persistent” (Boxall et al. 2004). Micropollutants, which include pharmaceuticals, pesticides, personal care products, disinfectants, and various other industrial chemicals, have been shown to impact aquatic ecosystems at different trophic levels from microbial communities (Burdon et al. 2020, Inostroza et al. 2025), macroinvertebrates (Beketov et al. 2013) to fish (Kidd et al. 2007, Parrish et al. 2019) and, therefore, can potentially result in structural and functional ecological alterations.

Microbial communities and particularly bacterial communities play an essential role in global biogeochemical cycling (Falkowski et al. 2008). They are the fundamental cornerstones of terrestrial and aquatic ecosystems. They facilitate various processes which mediate ecosystem services such as inorganic nutrient cycling, decomposition and mineralization of organic matter (Biasi et al. 2017) as well as the removal of natural and anthropogenic pollutants (Borreca et al. 2024). Furthermore, chronic exposure to antimicrobial substances can result in the evolution and dissemination of antimicrobial resistance genes within the ecosystem (Grenni et al. 2018).

Bacterial communities exhibit complex responses to micropollutants due to the site-specific community structures and their inherent functional redundancy. The majority of studies examining the effect of chemical pollution on riverine bacterial communities focussed on the impact of nutrients or heavy metals. Indeed, the impact of organic micropollutants on bacterial communities in surface waters has been analysed in only a few studies, usually in the context of wastewater-based emissions and/or by assessing pollution gradients at river basin level (Inostroza et al. 2025). Findings differs regarding effects on alpha-diversity with studies reporting no effects (Mansfeldt et al. 2020) or declined diversities (Burdon et al. 2020, Inostroza et al. 2025). However, the studies largely agree that chemical pollution causes significant changes of community composition (Gao et al. 2019, Burdon et al. 2020, Mansfeldt et al. 2020, Inostroza et al. 2025).

Similar trends emerge from studies that examined the impact of different land uses on bacterial communities in river ecosystems. While some studies revealed reduced diversities in bacterial communities inhabiting rivers exposed to agricultural and urban land use when compared to reference sites (Ibekwe et al. 2016, Chavarria et al. 2021, Li et al. 2023), others reported an increase in bacterial diversity in large urban areas (Gao et al. 2019, Zhao et al. 2021). Significant dissimilarities have been found in communities exposed to forested, agricultural, and urban land use (Chavarria et al. 2021, Fang et al. 2023). This highlights that shifts in bacterial community composition can be considered a more sensitive indicator that reflects different land-use types than alpha-diversity (Li et al. 2023).

Despite their essential role for ecosystem functioning, there is no standardised approach for assessing the impact of micropollutants on bacterial communities. Even within the European Union’s Water Framework Directive—the most significant European water legislation to date—only diatom biodiversity is considered as a microbial endpoint for assessing the biological quality of aquatic ecosystems. Bacterial communities are completely disregarded in the development of Environmental Quality Standards, which determine threshold concentrations for pollutants that should not be exceeded in order to preserve the ecological integrity of aquatic ecosystems (Pesce et al. 2020, Hellal et al. 2023).

Responses of bacterial communities to chemical pollution are assessed primarily based on environmental concentrations measured in water, which usually overlooks bioavailability and bioactivity of these micropollutants. Recently, Inostroza et al. (2025) applied minimum inhibitory concentrations (MICs) as threshold effect concentrations to normalise environmental concentrations of micropollutants following the “toxic unit” (TU) approach (Sprague 1970). This approach enables meaningful associations between bacterial responses and micropollutants. Furthermore, the combination of biogeochemical analyses with next-generation sequencing of DNA and/or RNA has the potential to comprehensively delineate shifts in bacterial communities. It also offers opportunities to explore relationships between bacterial diversity and function as well as how bacterial diversity and function are impacted by chemical.

The objectives of the study were to (i) characterise bacterial community structures in four small rivers using targeted-amplicon sequencing using environmental DNA (eDNA) in two seasons; (ii) determine associations between normalised micropollutant concentrations and bacterial community responses; and (iii) evaluate potential alterations in bacterial ecological functions of the bacterial microbiome due to micropollutants in the context of land use. Our efforts were focused on the Lake Victoria South basin (LVSB) in western Kenya (Africa), as a model in Africa for an area significantly affected by anthropogenic activities within the whole river basin. The microbial communities inhabiting this ecosystem are subjected to intensive agricultural activities, micropollutant discharges from untreated wastewater and sewage, small rural areas, and to some extent industrial activities associated with processing of crops (Tanui et al. 2024).

## Materials and methods

### Case study area—LVSB

The LVSB encompasses a catchment area of 26 906 km^2^ in the western part of Kenya. The LVSB is characterised by an equatorial hot and humid climate with a bi-modal rainfall pattern—long rains between March and May, and short rains from October to November. Between April and June all rivers in the basin exhibit a pronounced high runoff season (Aurecon AMEI Limited 2020). In the LVSB, land use is characterized by scattered urban and residential areas, forest, grassland/rangeland, and agricultural (mainly rain-fed crops such as maize, rice, and sugarcane) as well as industrial use, e.g. sugarcane factories (Tanui et al. 2024). The runoff from the LVSB commonly contains industrial effluents from major towns as well as municipal/domestic sewage from urban settlements. Additionally, untreated sewage is directly discharged into surface water systems as the local population is only partially connected to the sewer system. In addition, pesticide residues and nutrients from agriculture areas and agro-based industries end up in nearby waterbodies (Tanui et al. 2024).

**Figure 1.**
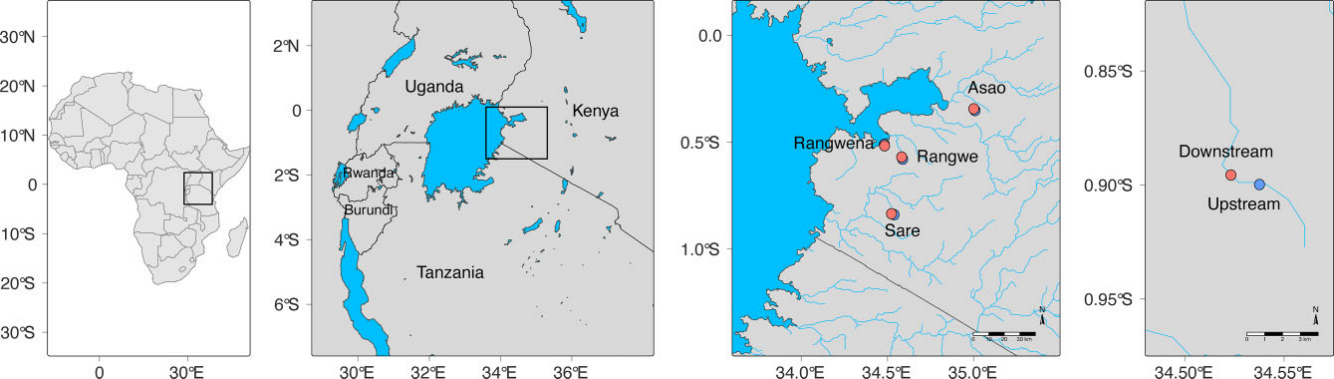
Location of the sampling sites though the LVS basin. (A) Sampling area in Africa. (B) Sampling area in the east part of Kenya. (C) Location of the sampling sites and the river’s names in the LVS basin. (D) Each river was sampled following an upstream and downstream strategy.

Four small rivers in the LVSB (Fig. 1) were selected based on the predominant land use (Supplementary Table 1) as follows: Firstly, river Rangwena flows close by an urban area, while passing through forests and areas with small-scale local farming of crops such as maize, cotton and tea. Consequently, the land use can be defined as a mix of natural, urban, and agricultural. River Asao is characterised by natural land use (forests and shrubland) and to a lower degree by semi-urban areas with small-scale local farming of crops; thus, its land use can be described as natural with urban and agricultural influence. River Rangwe flows through a semi-urban area where small-scale local farming of maize and livestock is conducted. Small forests can also be found in the vicinity. Therefore, the land use can be defined as a mix of agricultural and urban with some forested influence. Finally, River Sare passes through an urban area and large-scale agro-industrial sugarcane plantations and also receiving WWTP effluent from the sugarcane factory and untreated domestic waste; thus, the land use can be described as majorly urban with some agricultural influence (Linke et al. 2019).

### Physico-chemical parameters and nutrients

In addition to the eDNA samples, *i n situ* measurements of turbidity using WTW TURB 355 IR Turbidimeter, as well as conductivity, pH, temperature, and total dissolved solids using HANNA HI9811-5 were also performed. Additionally, 125 mL of grab water samples were collected, stored at 4°C and transported to the laboratory for measurements of carbonate hardness, phosphate-, nitrate, nitrite, and ammonium concentration, which were determined using semi-quantitative test strips (Quantofix, Merck).

### Sampling strategy for environmental DNA

In each river, two surface water samples were collected following an upstream—downstream setting (Fig. 1) in both wet (October 2021) and dry season (February 2022). Analysing both dry and wet seasons was essential to understand how agricultural practices, a key anthropogenic factor, contribute to chemical pollution. Farming in the region intensifies during the wet season, leading to a surge in the application of chemicals, including herbicides. Detailed information of the geographical locations of the selected sampling sites is provided in Supplementary Table 2. Sampling was performed by passing 200 to 500 mL of surface water per replicate through Sterivex® filters (Merck), depending on turbidity. All eDNA samples were immediately stored at −4°C in the field, before being transported to the lab and stored at −20°C until subsequent DNA extractions (Chiang and Inostroza 2022).

### DNA extraction protocols, sequencing, and bioinformatics pipelines

The DNA from water was extracted using the DNeasy PowerWater Kit (Qiagen, Germany) following an in-house protocol (Chiang and Inostroza 2022, Inostroza et al. 2025), which is a slightly modified version of the protocol provided by the manufacturer in order to improve DNA yields. The DNA samples were cleaned up and concentrated using AMPure XP clean-up kits (Beckman Coulter, USA) following the manufacturers protocol. Bacterial 16S rRNA genes were amplified utilising the V3-V4 primer pair (341F: CCTAYGGGRBGCASCAG; 806R: GGACTACNNGGGTATCTAAT) and sequenced using a 250-bp paired-end Illumina platform. PCR amplification and amplicon sequencing was conducted by Novogene Co. (Cambridge, UK). Eukaryotes were not included in the study due to a lack of genetic raw material, while archaea are insufficiently resolved by the used primer pair (McNichol et al. 2021).

Processing of the raw sequences was performed on resources provided by the Swedish National Infrastructure for Computing and National Academic Infrastructure for Supercomputing in Sweden at UPPMAX (Uppsala Multidisciplinary Center for Advanced Computational Science) at Uppsala University. Raw sequences were analysed using QIIME^TM^ 2 (Bolyen et al. 2019). The algorithm DADA2 was used for the denoising of the marker gene amplicon dataset (Knight et al. 2018). Within the algorithm, a maximum expected error of 2 and no truncation of the length of forward and reverse strand were set to achieve the best results. In addition, DADA2 utilised error profiles to resolve the sequence data into exact sequence features (ASVs). Afterwards, the SILVA database (Version: 138 SSURef NR99) was used as a reference to perform the taxonomic assignment and to construct a phylogenetic tree (Quast et al. 2013, Yilmaz et al. 2014). Pre-formatted, QIIME-compatible SILVA reference sequence and taxonomy files, provided by Robeson et al. (2021), were used for these tasks. Finally, Phylogenetic Investigation of Communities by Reconstruction of Unobserved States (PICRUSt2) was used to predict functional abundances of gene families (KEGG orthologs) based on the marker gene sequences in each sample (Douglas et al. 2020).

### Geospatial analysis

To support the analysis, the sample locations were matched to Pfafstetter level 12 sub-watershed polygons (15-arcsecond resolution) from the HydroATLAS database (Linke et al. 2019). HydroATLAS database was then used to retrieve corresponding land use and land cover in each individual polygon containing the samples (Supplementary Table 1). The selected spatial features are detailed in Supplementary Table 3. Geospatial analysis was conducted in ESRI ArcGIS Pro 3.1.0.

### Predicting antibiotic and antimicrobial stress (TU_MIC_ and TU_ECHA_)

Surface water samples were simultaneously grab sampled in all eDNA sampling sites. Measured environmental concentrations of micropollutants were published in a separate manuscript (Tanui et al. 2024). Effects on bacterial communities were assessed by normalising the concentrations of the antibiotics found (Supplementary Table 4) to their biological potency, as suggested by Inostroza et al. (2025). Antibiotic MICs were retrieved from a published database (Bengtsson-Palme and Larsson 2016). Besides, antimicrobial information was manually mined from the European Chemicals Agency (ECHA) database. The respective ECHA effect data were converted into chronic EC_10_-equivalents (effect concentration at which 10% effect is observed compared to the control group) following the recommendations of Warne et al. (2018). In both scenarios, a component-based approach was applied using the concentration addition (CA) mixture toxicity concept (Loewe and Muischnek 1926). Antibiotic stress (TU_MIC_) was calculated as in equation (1):


(1) \begin{eqnarray*}
\text{TU}_{MIC} = \sum\nolimits_{i = 1}^{n} \frac{{MEC}_{i}}{{MIC}_{i}}= \sum\nolimits_{i = 1}^{n} TU_{i},
\end{eqnarray*}

where MEC_i_ denotes the measured environmental concentration of the chemical *i*, while MIC_i_ represents the corresponding minimum inhibitory concentration of chemical *i*. The ratio MEC_i_/MIC_i_ provides a dimensionless measure of the individual toxicity contribution of each chemical present in the sample. Their sum is termed a “toxic unit” (TU) and describes the total antibiotic stress within the sample. The TU approach is prevalent in the risk assessment of chemical mixtures (e.g. pesticides, pharmaceuticals, industrial compounds, among others) on aquatic ecosystems (Inostroza et al. 2024, Weichert et al. 2025).

The lowest MIC was selected because antibiotic concentrations below the MICs have shown to select for resistant bacteria (Andersson and Hughes 2012, Gullberg et al. 2014). For instance, Gullberg et al. (2011) reported minimal selective concentrations—the minimum concentration of an antibiotic at which resistant bacterial strains possess a competitive advantage—to range between 1/230 and 1/4 of the corresponding MIC depending on the antibiotic.

Analogously, the antimicrobial stress exhibited by the group of chemicals derived from ECHA was termed TU_ECHA_, although, in this case, the individual chronic EC_10_ equivalent derived from ECHA of each chemical *i* was used for the calculation as in equation (2):


(2) \begin{eqnarray*}
\text{TU}_{ECHA} = \sum\nolimits_{i = 1}^{n} \frac{{MEC}_{i}} {EC10_{i}^{chronic}} = \sum\nolimits_{i = 1}^{n} TU_{i}.
\end{eqnarray*}

Both TU_MIC_ and TU_ECHA_ are indicators of antimicrobial stress lead by both antibiotics and antimicrobial chemicals, respectively.

The TU approach does not explicitly account for the bioavailability of chemicals in an environmental setting. The concentrations (MEC_i_) used in the TU calculations are based on total concentrations, that is dissolved and associated to particle form, whereas the effect concentrations (i.e. *MIC_i_* and *EC*10*_i_^chronic^*) are derived from standardised tests using simple media (total concentrations). Consequently, the TU_MIC_ and TU_ECHA_ models represent conservative approaches due to the use of total concentrations. Regarding the chemical’s bioactivity (here understood as their mode of action), the CA model is often applied to mixtures of micropollutants. This application is a pragmatic choice, not a perfect theoretical fit, although several studies support the use of the CA model in mixture risk assessment (Finckh et al. 2022, Inostroza et al. 2024, Weichert et al. 2025).

### Microbial community analysis

The ASV data were analysed using the R-package {phyloseq} (McMurdie and Holmes 2013). ASVs without taxonomic assignment at phylum level as well as eukaryotic, chloroplast-derived, and mitochondrial sequences were removed from the dataset (Knight et al. 2018), whereas singletons and doubletons were retained. The biological data were not rarefied, as suggested by McMurdie and Holmes (2014). For the analysis of alpha-diversity, five different metrics—Observed richness, Chao1, Shannon-Weaver, Pielou Evenness, and Faith´s phylogenetic distance were calculated for all samples. The non-parametric Wilcoxon-test was used to compare alpha diversities across different sampling sites, rivers, seasons, and environmental compartments. Furthermore, Venn diagrams were generated to visualize the number of unique and shared ASVs within each river and between seasons.

The Weighted Pair Group Method with Arithmetic Mean (WPGMA) was used to classify the different samples and to study their similarity. For beta-diversity, the ASV data were graphically represented using non-metric multidimensional scaling (NMDS). For the analysis weighted UniFrac was used as distance metric following the recommendations of Washburne et al. (2018). Dissimilarities were assessed between seasons as well as season-specific between rivers and sampling sites using Analysis of Similarity (ANOSIM), implemented in the {vegan package} (Martinez-Arbizu 2020).

Distance-based redundancy analysis (db-RDA) was used to analyse the relationship between beta-diversity and environmental parameters. For this purpose, the ASV data were first transformed using robust Aitchison distances, as suggested by Martino et al. (2019). On the other hand, the metadata, consisting of physico-chemical parameters, nutrients and antimicrobial stress (TU_MIC_ and TU_ECHA_), were transformed using Hellinger transformation according to Legendre and Gallagher (2001). Both datasets were linked in the db-RDA using weighted UniFrac as a distance metric. The Variance Inflation Factors (VIF) were initially calculated for the group of nutrients and physico-chemical parameters, respectively, in order to determine whether the constraining variables were redundant. All explanatory variables with a VIF score > 10 were excluded, while the remaining physico-chemical parameters and nutrients were grouped together with TU_MIC_ and TU_ECHA_. Redundant explanatory variables were removed from the analysis after additional VIF calculations to build the “best” db-RDA model. The significance of each non-redundant parameter was assessed using an Analysis of Variance (ANOVA, 500 permutations) and insignificant parameters were removed from the model. Finally, ANOVA (500 permutations) was conducted to assess the global significance of the “best” db-RDA model. The {vegan} R package was used to perform the db-RDA as well as all subsequent permutations tests (Oksanen et al. 2025).

Putative ecological function profiles were generated applying Functional Annotation of Prokaryotic Taxa (FAPROTAX) implemented in {microeco} (Liu et al. 2021). FAPROTAX is a database that maps prokaryotic clades (e.g. genera or species) to established metabolic or other ecologically relevant functions, using the current literature on cultured strains (Louca et al. 2016). We selected only ecological functions related to the ecosystem service degradation of toxic chemicals, and they were determined for each sample. Human and parasite related functions were not considered in our analysis.

Functional abundances of gene families, predicted by PICRUSt2, were mapped against the KEGG Orthology database (Kanehisa et al. 2023) to determine their associated functional pathways. Differential abundances/significant associations of functional pathways between rivers (within season) and sampling sites (within and between seasons) were analysed using Microbiome Multivariable Associations with Linear Models (MaAsLin2; normalization = TSS, correction = BH, transformation = AST; Mallick et al. 2021). We also focused on ecological functions related to the ecosystem service degradation potential of xenobiotics by bacterial organisms and thus we conducted seasonal comparisons, the predictions of upstream and downstream site were averaged for each putative ecological function and season, before calculating the ratio of wet to dry season. The ratio was determined for the whole bacterial community of each river.

The sequences for this study have been deposited in the National Center for Biotechnology Information under the project accession number PRJNA1152740.

## Results and discussion

The 16S rRNA amplicon sequencing provided a total of 1 991 397 raw sequences with an average of around 124 000 raw sequences per sample. The processing of the amplicon data resulted in a final dataset of 1 589 784 sequences including 6218 ASVs, 898 doubletons (14.4%), and 341 singletons (5.5%). Sufficient sequencing depth was reached in all samples (Supplementary Fig. 1). The taxonomic assignment, using the SILVA database as a reference, was successful for up to 80% and 30% of the taxa at genus and species level, respectively (Supplementary Fig. 2).

### Bacterial alpha-diversity and community composition

**Figure 2.**
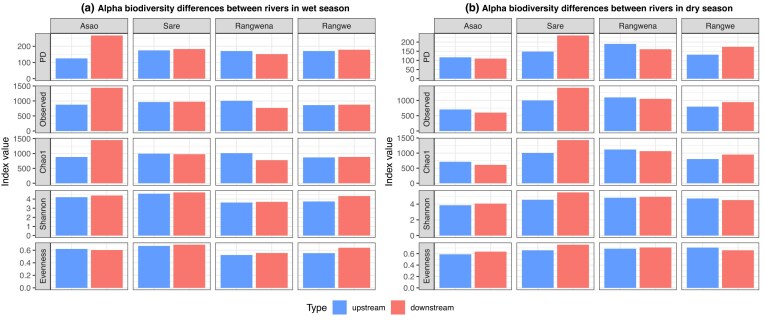
Alpha-diversity differences in surface water between rivers as boxplots in (A) wet and (B) dry seasons, collected in October 2021 and February 2022, respectively.

**Figure 3.**
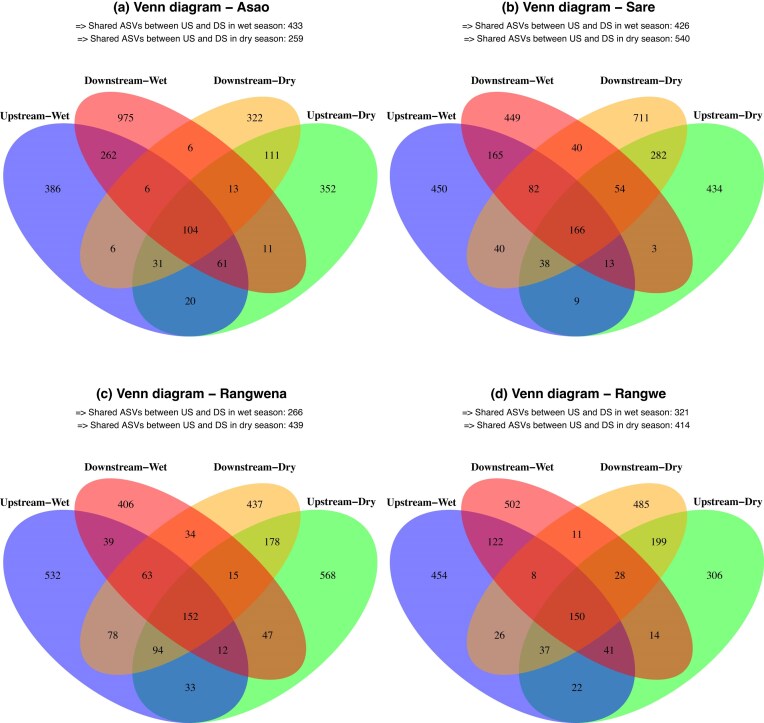
Venn diagrams showing the number of shared and unique ASVs for each river and season.

We observed an increase in alpha-diversity in downstream sites of the river Asao in the wet season and river Sare in the dry season (Fig. 2A and B). This is also supported by an increase of unique ASVs in downstream samples in Asao (from 386 to 975) and Sare (from 434 to 711) in wet and dry season, respectively (Fig. 3). Asao displayed up to three times more unique ASVs downstream compared to upstream conditions (Fig. 3A), while Sare showed a moderate increase of up to 1.5 times downstream regarding upstream (Fig. 3B). A plausible explanation is the input of novel taxa from runoff following heavy rains in the wet season in Asao and the higher relative amount of untreated domestic sewage effluents (containing its specific microbiota) in the dry season in Sare. In all other scenarios there were no clear differences in any alpha-diversity metric between upstream and downstream sites (Fig. 2A and B). Moreover, we did not observe significant differences in alpha-diversity between seasons across the rivers (Supplementary Fig. 3; Wilcoxon’s test, *P*-adjusted > 0.05).

An increase in alpha-diversity that correlates with urban influence in Sare during the dry season is consistent with previous studies featuring urban areas in their sampling areas (Gao et al. 2017, Zhao et al. 2021). Notably, these studies were conducted in larger urban areas compared to Sare, which is a small village with only a few hundred inhabitants. Nevertheless, at least one of the rivers flowing through these urban areas received untreated domestic sewage (Gao et al. 2017) similar to Sare. With respect to the seasonal variability, various studies support alpha-diversity increases in wet season in rivers influenced by urban (Gao et al. 2017, Fang et al. 2023), agricultural (Chen et al. 2018, Chavarria et al. 2021) or forested land use (Chavarria et al. 2021) as well as areas influenced by mixed land-use types (Wang et al. 2019, Laperriere et al. 2020, Chavarria et al. 2021, Shu et al. 2022), in line with our trend in Asao.

**Figure 4.**
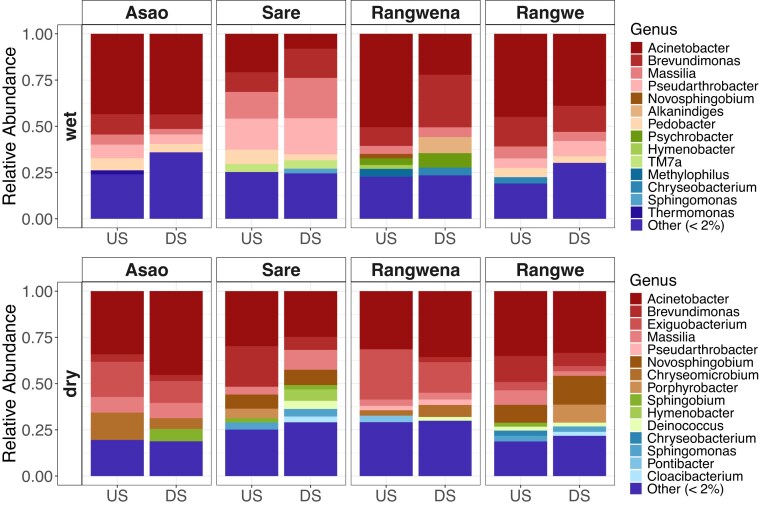
Relative community composition in surface water per river in wet and dry season collected in October 2021 and February 2022, respectively. Main phyla are color coded. US and DS represent the upstream and downstream sampling site, respectively.

Bacteria belonging to the genera *Acinetobacter* (up to 50%), *Brevundimonas* (up to 28%), *Massilia* (up to 22%), and *Pseudodarthrobacter* (up to 20%) dominated the bacterial composition in the studied rivers in the wet season (Fig. 4). In the dry season, the riverine bacterial community structures were mainly comprised of *Acinetobacter* (up to 45%), *Exiguobacterium* (up to 27%), *Brevundimonas* (up to 22%), *Novosphingobium* (up to 16%), and *Chryseomicrobium* (up to 15%) (Fig. 4). Interestingly, *Exiguobacterium* and *Chryseomicrobium* but also less abundant genera such as *Porphyrobacter, Sphingobium*, and *Deinococcus* were only detected during the dry season. In contrast, the genera *Alkanindiges, Pedobacter, Psychrobacter*, and *TM7a*, which exhibited peak abundances of 5%–9%, were only found in the studied rivers during the wet season. This indicates that there is a season-specific change in the bacterial community structures.

Sare, the river with the highest degree of urban land use, exhibited the most distinct community structure in the wet season displaying considerably lower abundances of *Acinetobacter* but higher abundances of *Masillia* and *Pseudarthrobacter* than the other rivers. Rangwena differed from Rangwe and Asao through the absence (*Pedobacter* and *Pseudarthrobacter*) and presence (*Novosphingobium, Psychrobacter, Hymenobacter*, and *Alkanindiges*) of less abundant genera. In addition, the downstream site of Rangwena displayed an increased abundance of *Brevundimonas* as well as fewer *Acinetobacter* than the other two rivers. In the dry season, the main differences between the rivers were higher abundances of *Brevundimonas, Porphyrobacter*, and *Novosphingobium* in rivers with a higher degree of urban land use (i.e. Sare and Rangwe), while Rangwena and Asao contained more *Exiguobacterium* and *Chryseomicrobium*. In fact, we found no *Exiguobacterium* in river Sare at all. However, Sare showed the most diverse community structure in the dry season, especially in the downstream site. In summary, rivers exposed to a high degree of natural land use (i.e. Asao and Rangwena) and a lower degree of urban influence (i.e. Asao, Rangwe, and Rangwena) showed high similarity in their community composition within the dry and wet season, respectively.

Many of the observed genera are either described as typical freshwater bacteria, including *Brevundimonas, Novosphingobium*, and *Porphyrobacter* (Zwart et al. 2002), or exhibit a cosmopolitan distribution in natural ecosystems—*Acinetobacter* (Jung and Park 2015), *Exiguobacterium* (Zhang et al. 2021), *Chryseobacterium* (Chaudhary and Kim 2017), and *TM7a* (Ferrari et al. 2014). However, the most abundant as well as season-specific genera have also been reported in freshwater ecosystems exposed to industrial, urban, and agricultural pollution (Yang et al. 2019, Laperriere et al. 2020, Liu et al. 2020, Zhang et al. 2020, Shao et al. 2021, Bohórquez-Herrera et al. 2023, Du et al. 2023).

Various genera play an important role in the degradation of natural and anthropogenic compounds including xenobiotics and thus, regulate biogeochemical fluxes and bioremediation in these riverine ecosystems. *Brevundimonas* (Zhang et al. 2025), *Chryseomicrobium* (Chaudhary and Kim 2017), *Massilia* (Selvarajan et al. 2024), and *Porphyrobacter* (Ding et al. 2025) can transform and degrade recalcitrant carbon, nitrogen, and phosphorous compounds thereby improving water quality. *Acinetobacter, Exiguobacterium, Novosphingobium, Sphingomonas*, and *Sphingobium* were found to be enriched after hydrocarbon pollution (Fuentes et al. 2015). Members of these groups are involved in the degradation of aromatic and hydrocarbon compounds (e.g. fuel oil) as well as xenobiotics (Stolz 2009, Newton et al. 2011, Chaudhary and Kim 2017, Yang et al. 2019, Yu et al. 2019, Kiama et al. 2021, Fang et al. 2023). Moreover, *Pseudarthrobacter* and *Chryseomicrobium* are capable of degrading PAHs and xenobiotics (Yu et al. 2019, Ghimire et al. 2024, Li et al. 2024). The widespread ability of xenobiotic degradation, especially of hydrocarbon compounds, suggests the pollution of these riverine ecosystems with substances such as petroleum.

The genera *Acinetobacter, Brevundimonas*, and *Massilia* contain opportunistic bacteria (Shao et al. 2021), whereas *Acinetobacter* and *Exiguobacterium* are also associated with antibiotic resistance (Jung and Park 2015, Fang et al. 2023). The high abundances of these genera in the four rivers indicates their exposure to untreated wastewater from rural settlements and urban areas.

Nonetheless, the abundances of many genera varied between seasons or different land uses. For instance, *Novosphingobium* has been described as an indicator for urban freshwaters (Laperriere et al. 2020). This agrees with its increased abundances in the rivers Sare and Rangwe in the dry season where the urban influence is higher due to a lack of rainfall. Furthermore, a seasonal change in the abundance of *Exiguobacterium* was previously observed (Fang et al. 2023). However, the study was conducted in an urban stream, while we did not detect this genus in the urban river Sare but had generally higher levels in agricultural streams. Lastly, the genus *Pseudarthorbacter* is typically enriched in petroleum-contaminated soils (Ghimire et al. 2024, Li et al. 2024) but has also been detected in rivers exposed to urban pollution (Zhang et al. 2020). The elevated abundances of these bacteria in all four rivers in the wet season could be explained by the increased rainfall in that month and thus, runoff from potentially petroleum-contaminated soils into the rivers.

### Beta-diversity and relationship with environmental parameters

**Figure 5.**
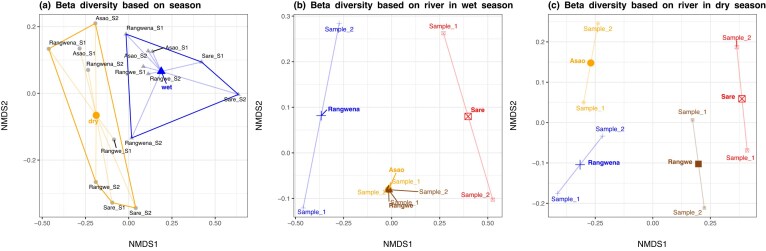
Differences in beta-diversity between (A) seasons (stress < 0.1) as well as between rivers in wet season (stress < 0.0001) and (C) dry season (stress < 0.03). Weighted UniFrac was used as a distance metric. US and DS represent the upstream and downstream sampling site, respectively.

We identified significant seasonal differences between the bacterial communities (Fig. 5A; PERMANOVA test, *P*-adjusted = 0.001) as well as between the rivers in both wet (ANOSIM, *P* = 0.01, *R* = 0.6) and dry (ANOSIM, *P* = 0.01, *R* = 0.8) season (Fig. 5B and C). These observations are consistent with previous studies finding seasonal dissimilarities in communities exposed to forested or agricultural land use (Chavarria et al. 2021) and highly urbanised and densely populated areas (Fang et al. 2023). Furthermore, several studies reported significant differences between seasons in large areas impacted by mixed land use (Liu et al. 2023a, Sun et al. 2021, Zhao et al. 2021, Shu et al. 2022). Moreover, the NMDS shows a clear clustering of rivers in both seasons which was further supported by the WPGMA cluster tree (Supplementary Fig. 4).

While in the dry season the rivers were grouped based on their high (i.e. Asao and Rangwena) and low (i.e. Rangwe and Sare) degree of natural land use, the high similarity of Asao and Rangwe in the wet season can be explained by the elevated input of agricultural and/or forested runoff due to the heavy rains. Previous studies support the finding that forests and farmland are the dominant land-use types to shape the bacterial community at sub-basin scale (Shu et al. 2022). Especially in the dry season, forested areas are impacting the community structure through the input of non-freshwater bacteria (Zhao et al. 2021). Nonetheless, our results also highlight the role of small, urbanised areas (i.e. Sare) as factors affecting the structure of bacterial communities similar to larger urbanised areas. Finally, all three land-use types—forested, urban, and agricultural—and their combinations are known to impact bacterial community structure in rivers enhancing bacterial dissimilarities (Liu et al. 2023a, Wang et al. 2016, Laperriere et al. 2020, Liao et al. 2020, Chavarria et al. 2021, Inostroza et al. 2025).

**Figure 6.**
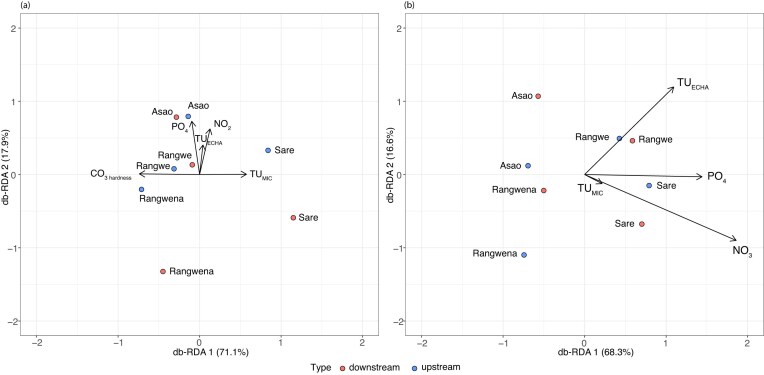
Db-RDA for rivers in (A) wet and (B) dry seasons. Significant models were built on five or four environmental variables, respectively. Significant environmental variables were water hardness (CO_3__hard) during the wet season and nutrients (PO_4_^3−^ and NO_3_^−^) together with toxic stress from antimicrobial chemicals (TU_ECHA_/TU_MIC_) during the dry season.

We analysed three sets of environmental parameters using db-RDA, in order to evaluate their impacts on the structure of bacterial communities: nutrients, physico-chemical parameters, and the toxic stress exhibited by organic chemicals with antimicrobial properties (Supplementary Table 5). The analysis revealed that the observed dissimilarities between the rivers was best explained by carbonate hardness, nutrients, TU_MIC_ and TU_ECHA_ (Fig. 6). Specifically, our findings show a positive correlation antibiotic stress (TU_MIC_) the urban river Sare in wet and dry seasons (Fig. 6A and B). Phosphate and nitrite together with antimicrobial stress (TU_ECHA_) correlated with streams receiving elevated agricultural runoff (i.e. Asao and Rangwe) in the wet season (Fig. 6A). Interestingly, in the dry season, phosphate and nitrite correlate together with TU_MIC_ affecting the urban river Sare (Fig. 6B). Sare is the only site primarily affected by urban areas and the discharge from a WWTP. Our findings support the role of antibiotics discharged from WWTPs affecting the structure and potential functioning of bacterial communities through the TU_MIC_ indicator. Similar pattern has been determined in small rivers affected by antibiotics in South America applying the same antibiotic stress indicator (Inostroza et al. 2025).

We determined that metronidazole, oxytetracycline, and ciprofloxacin were the main drivers responsible of the predicted antibiotic stress (TU_MIC_). These antibiotics contributed to the overall stress in a ratio of 0.61, 0.93, and 0.96 (Supplementary Table 5), respectively. Our findings suggest that few chemicals may be responsible for the structural dissimilarities within the bacterial communities. A similar pattern was observed by Inostroza et al., (2025) in a river basin study conducted in South America (Chile).

Previous studies support the significant role of nutrients in shaping the bacterial community structure both in agricultural (Liu et al. 2023b) and urban rivers (Gao et al. 2017, Fang et al. 2023). However, in contrast to our findings, the impact of nutrients was not restricted to a specific season but was observed across all seasons for each land-use type. Furthermore, we did not observe a significant role of physico-chemical parameters such as pH, conductivity, temperature or flow rate in forested, urban or agricultural rivers unlike previous studies (Liu et al. 2023b, Wang et al. 2016, Gao et al. 2017, Chavarria et al. 2021).

### Potential ecological changes in bacterial communities

**Figure 7.**
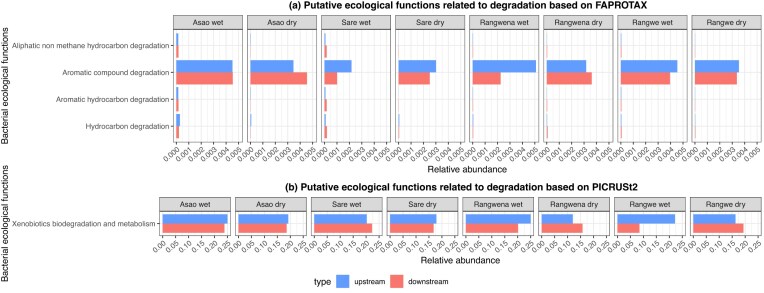
Putative ecological functions of the whole bacterial community related to degradation for each river and season, predicted by (A) FAPROTAX and (B) PICRUSt2. Sampling sites are color coded.

We assessed potential shifts in bacterial ecological functions in response to land-use and seasonal variations within the LVS basin. We focused on relevant bacterial ecological functions contributing to essential ecosystem services linked to biodegradation of chemicals in the environment, including degradation of aliphatic non-methane hydrocarbons, aromatic compounds, aromatic hydrocarbons, hydrocarbons, and xenobiotics (Fig. 7).

Overall, aromatic compound degradation was the most abundant function predicted by FAPROTAX, showing elevated relative abundances of bacteria featuring this ecological function (up to 50%) in forested and agricultural rivers (e.g. Asao, Rangwena, and Rangwe). The dynamics of this metabolic function across seasons and different land uses (Fig. 7) seem to follow the dynamics of the relative abundance of the genus *Acinetobacter* (Fig. 4). For instance, this genus had the lowest abundances in the urban river Sare which is a plausible explanation why Sare also had the lowest predicted values for aromatic compound degradation. The higher abundance of this ecological function, compared to other studies analyzing similar degradation functions in other river systems (Yao et al. 2022, Inostroza et al. 2025), potentially indicates pollution of Kenyan rivers with substances commonly found in fossil fuels such as petrol as well as pharmaceuticals and pesticides. The other three pathways (aliphatic non methane hydrocarbon, aromatic hydrocarbon, and hydrocarbon degradation) displayed comparatively low abundances across the board. Metabolic pathways related to degradation of xenobiotic chemicals were determined with high relative abundances (up to 25%) across all rivers but especially in Asao and Sare. This is consistent with previous studies that detected high concentrations of different xenobiotics in Kenyan freshwater systems (Tanui et al. 2024).

PICRUSt2 predicted the potential occurrence of bacteria capable of degrading xenobiotics. The dynamics of metabolic pathways related to xenobiotic degradation across seasons and land use are difficult to attribute to specific community members. The ability to degrade xenobiotics is widespread across the different genera including *Acinetobacter* (Jung and Park 2015), *Exiguobacterium* (Kiama et al. 2021, Selvarajan et al. 2024), *Chryseomicrobium* (Chaudhary and Kim 2017, Yu et al. 2019), *Pseudarthrobacter* (Ghimire et al. 2024, Li et al. 2024), *Novosphingobium, Sphingomonas*, and *Sphingobium* (Stolz 2009, Newton et al. 2011). Our findings indicate an increase of bacterial degradation potential in the wet season (Table 1). Similar enrichment patterns have been observed in surface waters in large urban areas in the dry season (Fang et al. 2023).

**Table 1.** Comparison of the xenobiotic degradation potential—predicted by FAPROTAX or PICRUSt2—of each river´s whole bacterial community between wet and dry season.

	Ratio between wet and dry season			
	Asao	Sare	Rangwena	Rangwe
Aliphatic non-methane hydrocarbon degradation (FAPROTAX)	8.7	23	1.3	2.4
Aromatic compound degradation (FAPROTAX)	1.1	0.58	1.1	1.2
Aromatic hydrocarbon degradation (FAPROTAX)	8.7	23	1.3	2.4
Hydrocarbon degradation (FAPROTAX)	5.7	2.9	0.95	1.9
Xenobiotics biodegradation and metabolism (PICRUSt2)	1.2	1.2	1.4	0.90

Upstream and downstream site were averaged, before calculating the seasonal ratio. Ratios greater than 1 indicate an increased degradation potential in the wet season, while the opposite is the case if the ratio is lower than 1.

The consistently high potential for aromatic compound and xenobiotic degradation within the riverine bacterial communities across seasons and different land-use types implies a chronic or recurrent exposure to these compounds. While the bacterial community structure varied between rivers and seasons, the overall metabolic capacity for the removal of environmental pollutants was maintained—likely due to the presence of functionally redundant and metabolically versatile taxa (Ramond et al. 2025). The higher bacterial degradation potential in the wet season likely results from increased inputs of pollutants into the rivers through urban and agricultural runoff thereby changing bacterial community composition or enhancing microbial activity. Overall, these patterns point to a resilient and adaptable bacterial community capable of sustaining their ability to degrade xenobiotics under varying environmental conditions and anthropogenic stressors.

## Conclusions

In summary, this study assessed the structural and potential functional responses of bacterial communities to co-occurring antimicrobial pollutants, present at trace concentrations (ng/L), in the LVSB (western Kenya). We included available antimicrobial effect information available from ECHA together with MIC information in order to assess how micropollutants impact bacterial communities on a structural and functional level.

Our findings reveal that bacterial community composition effectively reflects the impact of chemical pollution measured in small rivers with distinct land uses. Although trends in alpha-diversity were noted, beta-diversity exhibited significant variation within each season between rivers flowing through small, urbanized areas (e.g. Sare) and rivers primarily exposed to forested areas and/or agricultural activities (e.g. Asao, Rangwena, and Rangwe). This seasonal variation was best explained by exposure to nutrients and antimicrobial stress (normalized concentrations of antibiotic and antimicrobial micropollutants). Furthermore, the functional analysis showed a generally high potential for aromatic compound and xenobiotic degradation within all bacterial communities but revealed a season-specific reduction in bacterial degradation potential across all four rivers.

Our results indicate that the impact of urbanised areas characterised by subsistence agriculture on bacterial biodiversity is similar to that observed in areas with a high degree of urbanisation, intensive agriculture, or mixed land use in other parts of the world.

## Supplementary Material

fnaf113_Supplemental_File

## Data Availability

Data is available in open repositories following FAIR Data Principles in the National Center for Biotechnology Information (NCBI) under the project accession number PRJNA1152740.
