# Supplementary material for: Bacterial community responses to micropollutants in chemically stressed small rivers in Kenya using environmental DNA
Source: FEMS Microbiol Lett. 2025 Oct 16;372:fnaf113. doi: 10.1093/femsle/fnaf113 (PMC12598650; doi:10.1093/femsle/fnaf113)
Supplement: fnaf113_Supplemental_File [file fnaf113_supplemental_file.docx]

**Supplementary material**

**Bacterial community responses to micropollutants in chemically-stressed small rivers in Kenya using environmental DNA**

Nicolai Verbücheln^1,2^, Sonja Schaufelberger^1^, Tibaud Cardis^2^, Isaac C. Tanui^3,4,5^, Faith Kandie^6,7^, Werner Brack^3,4^, Thomas Backhaus^1,8^, Pedro A. Inostroza^1,8,*^

^1^ Department of Biological and Environmental Sciences, University of Gothenburg, Gothenburg, Sweden

^2^ Department of Marine Sciences, University of Gothenburg, Gothenburg, Sweden

^3^ Department of Exposure Science, Helmholtz Centre for Environmental Research (UFZ), Leipzig, Germany

^4^ Institute of Ecology, Evolution and Diversity—Goethe University, Frankfurt am Main, Germany

^5^ Department of Chemistry and Biochemistry, Moi University, Eldoret Kenya

^6^ Department of Biological Sciences, Moi University, Eldoret Kenya

^7^ Stellenbosch Institute for Advanced Study, Stellenbosch, South Africa

^8^ Institute for Environmental Research, RWTH Aachen University, Aachen, Germany

* Corresponding author: Dr. Pedro A. Inostroza, Institute for Environmental Research, RWTH Aachen University, Worringerweg 1, 52074 Aachen, Germany. E-mail: pedro.inostroza@rwth-aachen.de

| **** |
| --- |
| Supplementary Figure 1. Rarefaction curves for each sample. Rivers are colour coded. |

| **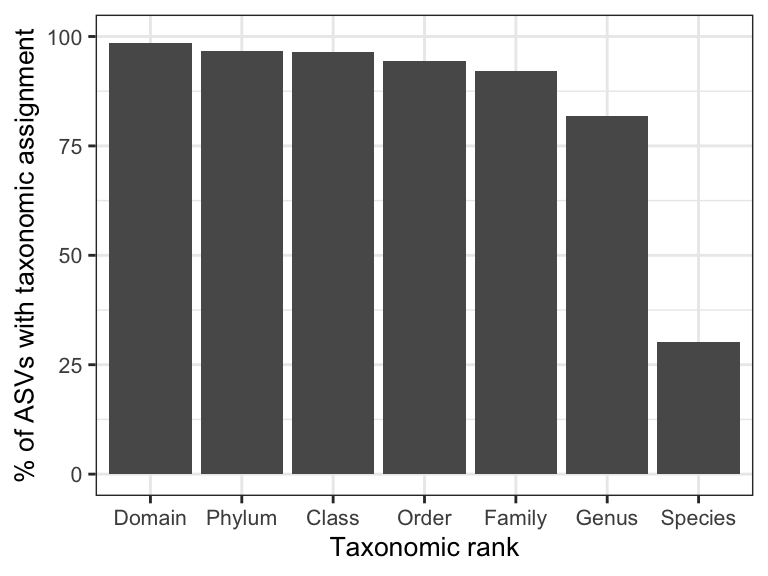** |
| --- |
| Supplementary Figure 2. Performance of the bacteria taxonomic assignment using SILVA as a reference database. |

| 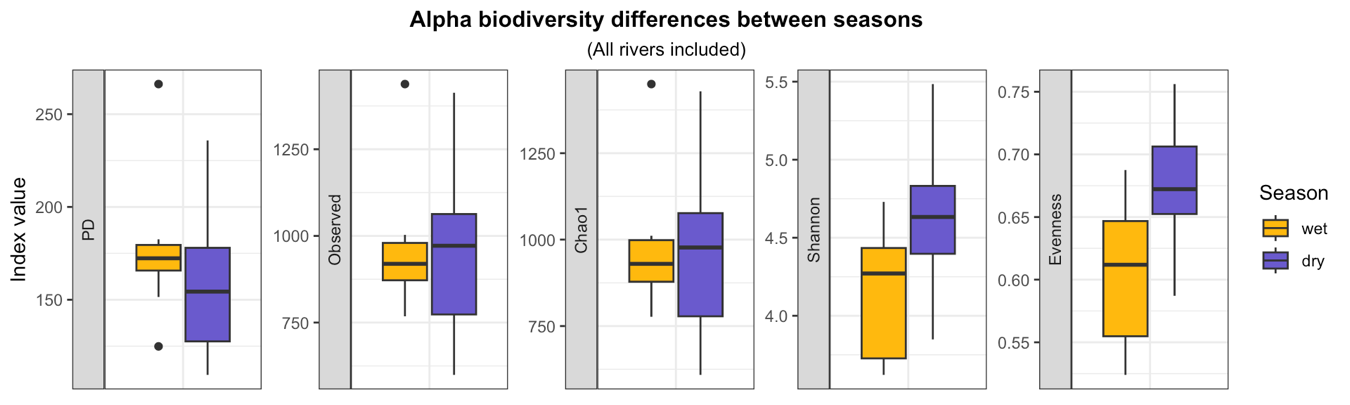 |
| --- |
| Supplementary Figure 3. Alpha biodiversity differences in surface water between wet and dry season collected in October 2021 and February 2022, respectively. Seasons are colour coded. |

| **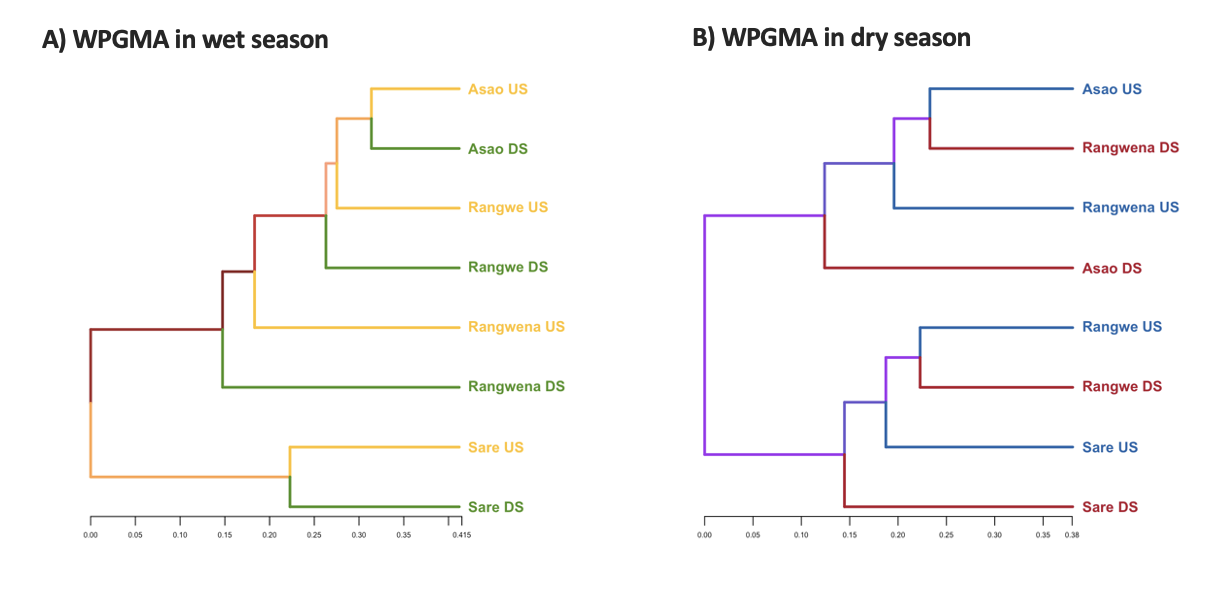** |
| --- |
| Supplementary Figure 4. WPGMA cluster tree for bacterial communities sampled during A) wet and B) dry season using weighted UniFrac distances. US and DS represent the upstream and downstream sampling site, respectively. Sampling sites are colour coded for each season. |

Supplementary Table 1. Land-use data from geospatial analysis

| **River** | **Total area [km^2^]** | **Tree cover [%]** | **Grass cover [%]** | **Shrubs cover [%]** | **Agriculture cover [%]** | **Urban cover [%]** | **Bareland cover [%]** |
| --- | --- | --- | --- | --- | --- | --- | --- |
| **Rangwena** | 146.9 | 31 | 0 | 2 | 20 | 24 | 0 |
| **Rangwe** | 180 | 8 | 0 | 0 | 36 | 28 | 0 |
| **Asao** | 152.8 | 31 | 0 | 9 | 12 | 18 | 0 |
| **Sare** | 143.9 | 0 | 0 | 0 | 19 | 58 | 0 |

Supplementary Table 2. Sampling site information. Geographical coordinates in decimal degree (WGS84).

| River | Site | Sample date | Latitude | Longitude |
| --- | --- | --- | --- | --- |
| Rangwena | DS | 2021-10-07  2022-02-10 | -0.519796 | 34.482900 |
| Rangwena | US | 2021-10-07  2022-02-10 | -0.511802 | 34.481537 |
| Rangwe | DS | 2021-10-03  2022-02-12 | -0.582528 | 34.582272 |
| Rangwe | US | 2021-10-03  2022-02-12 | -0.593827 | 34.587787 |
| Asao | DS | 2021-10-04  2022-02-07 | -0.313919 | 35.000071 |
| Asao | US | 2021-10-04  2022-02-07 | -0.323101 | 35.007005 |
| Sare | DS | 2021-10-05  2022-02-11 | -0.898537 | 34.523174 |
| Sare | US | 2021-10-05  2022-02-11 | -0.903089 | 34.537552 |

Supplementary Table 3. Land-cover and land-use variables retrieved from HydroATLAS.

| Data | Unit | Reference |
| --- | --- | --- |
| Land-cover |  |  |
| Total area | km2 | (Linke et al., 2019) |
| Bare cover | % | (Bartholomé and Belward, 2005) |
| Grass cover | % | (Bartholomé and Belward, 2005) |
| Shrubs cover | % | (Bartholomé and Belward, 2005) |
| Tree cover | % | (Bartholomé and Belward, 2005) |
| Land-use |  |  |
| Cropland extent | % | (Ramankutty et al., 2008) |
| Urban extent | % | (Pesaresi and Freire, 2016) |

Supplementary Table 4. Measured environmental concentrations of antibiotics and antimicrobials in the sampled rivers.

| Antimicrobials | Rangwena_US_WET | Rangwena_DS_WET | Rangwe_US_WET | Rangwe_DS_WET | Asao_US_WET | Asao_DS_WET | Sare_US_WET | Sare_DS_WET | Rangwena_US_DRY | Rangwena_DS_DRY | Rangwe_US_DRY | Rangwe_DS_DRY | Asao_US_DRY | Asao_DS_DRY | Sare_US_DRY | Sare_DS_DRY |
| --- | --- | --- | --- | --- | --- | --- | --- | --- | --- | --- | --- | --- | --- | --- | --- | --- |
| 2-Octyl-4-isothiazolin-3-one |  | 0.6 | 0.6 | 0.4 | 0.6 | 0.6 | 0.5 |  |  |  |  |  |  | 0.5 |  |  |
| 4-Hydroxyquinoline | 11.4 | 2.2 |  | 2.5 | 6.3 |  | 3.3 | 7.2 |  |  | 3.2 | 2.0 | 2.7 | 1.8 | 1.4 |  |
| Acetyl-sulfamethoxazole | 91.8 | 25.4 | 4.7 | 69.9 | 11.0 | 11.7 | 4.6 | 26.7 | 12.0 | 21.9 | 178.1 | 68.0 | 717.2 | 21.0 | 18.4 | 16.5 |
| Benzyldimethyldecylammonium |  |  |  |  | 9.3 |  |  |  |  |  |  |  |  |  |  |  |
| Benzyldimethyldodecylammonium |  |  |  |  | 103.7 |  |  |  |  |  |  |  |  |  |  |  |
| Benzyldimethylhexadecylammonium |  |  |  |  | 4.6 |  |  |  |  |  |  |  |  |  |  |  |
| Chloramphenicol |  |  |  |  |  |  |  |  |  |  |  | 5.7 |  |  |  |  |
| Clotrimazole | 9.6 |  |  |  |  |  |  |  |  |  |  |  |  |  |  |  |
| Diclosan | 4.4 | 1.8 | 2.3 |  |  |  |  |  | 7.0 | 1.6 | 2.5 |  |  |  |  |  |
| Erythromycin |  |  |  |  | 8.3 |  |  |  |  |  |  |  |  |  |  |  |
| Ethylparaben | 1.6 | 2.7 | 2.2 | 0.5 | 4.9 | 0.5 | 3.9 | 1.6 |  |  |  |  |  |  |  |  |
| Fluconazole | 3.0 | 1.9 |  |  | 3.5 | 2.8 |  | 4.3 | 3.1 | 9.1 | 10.2 | 11.2 | 13.0 | 6.2 |  | 2.1 |
| Mebendazole |  |  | 0.3 | 0.3 | 0.3 |  |  |  |  |  | 0.4 | 0.5 | 0.6 |  |  |  |
| Methylparaben | 39.0 |  | 19.9 |  | 18.4 |  | 21.5 |  |  |  |  |  |  |  |  |  |
| Picoxystrobin |  |  |  | 5.3 |  |  |  |  | 58.0 |  |  |  |  |  |  |  |
| Propylparaben | 6.2 |  |  |  |  |  | 6.5 |  |  |  | 2.3 |  |  | 2.0 |  |  |
| Sulfamethoxazole | 25.0 | 7.2 | 3.3 | 32.7 | 8.9 | 6.6 | 2.4 | 8.8 | 4.6 | 9.7 | 109.1 | 53.5 | 59.4 | 16.2 | 8.4 | 8.3 |
| Triclosan |  |  | 1.9 | 3.4 |  |  |  |  |  |  |  | 1.1 |  |  |  |  |
| Trimethoprim | 41.8 | 17.0 | 9.5 | 3.9 | 41.2 | 3.8 |  |  | 15.4 | 10.6 | 85.2 | 24.3 | 882.2 | 7.4 | 2.3 | 5.2 |

Supplementary Table 5. Metadata and measured environmental parameters.

| River | Type | Season | Temp  [°C] | Cond  [μS] | pH | DO  [mg/L] | Flow  [m/s] | turb | PO_4_  [mg/L] | NO_3_  [mg/L] | NO_2_  [mg/L] | CO_3__hard  [°d] | TU_ECHA_ | TU_MIC_ |
| --- | --- | --- | --- | --- | --- | --- | --- | --- | --- | --- | --- | --- | --- | --- |
| Rangwena | DS | dry | 22.7 | 770 | 7.10 | 0.98 | 0.10 | 332.80 | 10,00 | 25.00 | 0.50 | 20.00 | 0.0000037 | 0.00484 |
| Rangwena | DS | wet | 21.8 | 990 | 7.30 | 10.35 | 1.25 | 115.50 | 10,00 | 20.00 | 0.25 | 20.00 | 0.0000040 | 0.00845 |
| Rangwena | US | dry | 26.4 | 820 | 6.50 | 6.30 | 0.25 | 158.90 | 10,00 | 25.00 | 0.50 | 20.00 | 0.0000019 | 0.00372 |
| Rangwena | US | wet | 25.8 | 930 | 7.40 | 3.62 | 1.60 | 60.99 | 10,00 | 20.00 | 0.25 | 20.00 | 0.0000075 | 0.01352 |
| Rangwe | DS | dry | 20.2 | 150 | 6.70 | 6.79 | 0.70 | 129.60 | 3,00 | 10.00 | 0.50 | 6.00 | 0.0000102 | 0.00798 |
| Rangwe | DS | wet | 20.4 | 120 | 6.70 | 8.13 | 2.10 | 113.90 | 3,00 | 2.50 | 0.25 | 6.00 | 0.0000091 | 0.00177 |
| Rangwe | US | dry | 24.1 | 140 | 6.40 | 7.95 | 0.95 | 103.00 | 10,00 | 10.00 | 0.50 | 6.00 | 0.0000072 | 0.01814 |
| Rangwe | US | wet | 24.6 | 110 | 6.80 | 8.48 | 2.15 | 101.00 | 3,00 | 2.50 | 0.25 | 6.00 | 0.0000030 | 0.00196 |
| Asao | DS | dry | 23.5 | 720 | 7.50 | 7.77 | 0.10 | 5.23 | 10,00 | 2.50 | 0.25 | 20.00 | 0.0000049 | 0.00282 |
| Asao | DS | wet | 20.3 | 200 | 6.80 | 8.49 | 5.00 | 47.33 | 10,00 | 2.50 | 0.25 | 6.00 | 0.0000013 | 0.00420 |
| Asao | US | dry | 24.4 | 490 | 7.50 | 6.37 | 2.05 | 5.93 | 10,00 | 2.50 | 0.25 | 20.00 | 0.0000084 | 0.11653 |
| Asao | US | wet | 26.6 | 170 | 7.50 | 8.40 | 2.45 | 64.58 | 10,00 | 2.50 | 0.25 | 6.00 | 0.0000149 | 0.00792 |
| Sare | DS | dry | 19.1 | 90 | 6.90 | 7.83 | 1.35 | 113.80 | 10,00 | 10.00 | 0.50 | 6.00 | 0.0000029 | 0.04550 |
| Sare | DS | wet | 18.6 | 70 | 6.60 | 7.81 | 2.85 | 336.90 | 3,00 | 2.50 | 0.25 | 6.00 | 0.0000049 | 0.02072 |
| Sare | US | dry | 25.1 | 90 | 6.00 | 8.18 | 2.15 | 69.06 | 10,00 | 10.00 | 0.25 | 6.00 | 0.0000015 | 0.00067 |
| Sare | US | wet | 19.0 | 60 | 5.90 | 7.71 | 2.95 | 441.00 | 10,00 | 2.50 | 0.25 | 6.00 | 0.0000042 | 0.00834 |
